# Detection of *Clostridioides difficile* toxin B gene: benefits of identifying gastrointestinal pathogens by mPCR assay in the diagnosis of diarrhea in pediatric patients

**DOI:** 10.1186/s12879-022-07104-z

**Published:** 2022-02-05

**Authors:** Jung-Hyun Byun, Dongeun Yong, Heejung Kim

**Affiliations:** 1 Department of Laboratory Medicine, Gyeongsang National University Hospital, Gyeongsang National University School of Medicine, Jinju, South Korea; 2 Department of Laboratory Medicine and Research Institute of Bacterial Resistance, Severance Hospital, Yonsei University School of Medicine, Seoul, South Korea; 3 Department of Laboratory Medicine and Research Institute of Bacterial Resistance, Severance Hospital, Yonsei University School of Medicine, Yongin, Gyeonggi-do South Korea

**Keywords:** *Clostridioides difficile*, Intestinal colonization, CDI, Toxin, Bbacterial diarrhea

## Abstract

**Background:**

In the pediatric population, severe *Clostridioides difficile* infection (CDI) sometimes occurs, but most cases are asymptomatic. The asymptomatic carriage rate in pediatric populations is reportedly higher than in the adult population. It is difficult to diagnose CDI, even if *C. difficile* is detected in children with diarrhea. This study aimed to evaluate the positivity rate of toxigenic *C. difficile* in the pediatric population with diarrhea.

**Methods:**

We collected and retrospectively analyzed gastrointestinal pathogen multiplex PCR results of 960 patients to estimate the positivity rate of toxigenic *C. difficile* in pediatric populations aged between 0 and 18 years.

**Results:**

The overall rate of *C. difficile* toxin B positivity was 10.1% in the stool samples. The positivity rate peaked in 1-year-old infants (29/153, 19.0%) and continually decreased thereafter. The positivity rate we observed was lower than the rates described in the literature. Remarkably, no *C. difficile* was detected in neonates. Antibiotic usage was inversely related to the positivity rate, especially in infants < 2 years of age. The odds ratio of antibiotics was 0.44 (95% confidence interval (CI) 0.28–0.68; *P* < 0.001). The presence of concomitant gastrointestinal pathogens was not associated with toxigenic *C. difficile* positivity.

**Conclusions:**

Even though toxigenic *C. difficile* infection is neither an important nor a common cause of pediatric diarrhea, children can spread it to adults at risk of developing CDI. The pediatric population can act as hidden reservoirs for pathogenic strains in the community.

## Background

*Clostridioides difficile*, formerly known as *Clostridium difficile*, is a spore-forming, obligate anaerobic, gram-positive bacillus acquired either from the environment or through the fecal-oral route [1]. It is known to cause a wide range of symptoms, from mild diarrhea to severe life-threatening complications such as toxic megacolon [2]. The major virulence factors of *C. difficile* are large clostridial toxins, toxin A and toxin B, which are encoded by *tcd*A and *tcd*B [3]. *C. difficile* infection (CDI) mainly occurs in healthcare-associated cases and adults. However, over the last decade, CDI has emerged as an important community-associated infection both in adults and children [4]. Approximately 4–5% of non-hospitalized healthy adults carry the pathogen in their intestinal flora [5], and varying positive rates of up to 70% have been reported in healthy newborns [6]. In children, the carrying capacity decreases with age, reaching adult levels of approximately 5% by the age of 2 [7]. According to the guidelines for pediatric consideration, because of the high prevalence of asymptomatic carriage of toxigenic *C. difficile*, testing for CDI should not be routinely performed in children under 12 months of age with diarrhea [1, 8]. If they have rare and severe symptoms of pseudomembranous colitis, toxic megacolon, or clinically significant diarrhea, *C. difficile* testing should be performed. In children aged between 1 and 3 years, a diagnostic workup for other diarrheal causes should be performed first, while *C. difficile* testing can be considered at later stages. Due to the unclear role of *C. difficile* in children with diarrhea, there are few reports on the positivity of toxigenic *C. difficile* in the pediatric population. Therefore, this study focuses on multiplex PCR (mPCR), which is increasingly being applied to detect gastrointestinal pathogens and provides additional information on the *C. difficile* toxin B gene (*tcd*B) in pediatric patients. In this study, we estimated the positivity rate of *C. difficile tcd*B and interpreted the results through electronic medical record review.

## Methods

We reviewed 960 non-duplicated stool mPCR (Seeplex Diarrhea-B1/2 and V ACE Detection, Seegen, Korea) results obtained from pediatric patients up to the age of 18 [9] collected over 39 months (October 2014–December 2017) and submitted to a tertiary referral hospital in Seoul. The mPCR included *C. difficile tcd*B and 13 other diarrhea-causing pathogens (*Salmonella* spp., *Shigella* spp., *Vibrio* spp., *Campylobacter* spp., *Escherichia coli* O157:H7, *Clostridium perfringens, Yersinia enterocolitica, Aeromonas* spp., Verocytotoxin-producing *E. coli*, rotavirus group A, norovirus, astrovirus, and enteric adenovirus). The electronic medical records of the patients were reviewed to acquire information regarding age, length of hospital stay, underlying diseases (malignant neoplasm, hematology/immunology, endocrinology, cardiovascular, respiratory, digestive, inflammatory bowel diseases, and genitourinary disorder), previous history of antibiotics, and clinical diagnosis of *C. difficile* enterocolitis during the entire period of hospitalization with or without metronidazole or oral vancomycin treatment.

Unpaired *t*-test or Mann-Whitney U test was used for continuous data. Pearson’s chi-squared test or Fisher’s exact test was used for categorical data. The odds ratios of the antibiotic-treated versus naive groups were calculated. All statistical analyses were performed using MedCalc Statistical Software version 18 (MedCalc Software bvba, Ostend, Belgium; http://www.medcalc.org; 2018).

## Results

### ***Clinical characteristics of C. difficile tcd*B positivity and*tcd*B negativity**

The overall positivity of *C. difficile tcd*B, as determined by mPCR, was 10.1% (97/960). Patients with positive results were younger (median age, 1.6 years) than those with negative results (median age, 3.8 years) (*P* <0.01) (Table 1).

**Table 1:** Baseline characteristics of 960 pediatric patients with diarrhea based on *Clostridioides difficile* toxin B (*tcd*B) detected by multiplex PCR

	*tcd*B positive (N = 97)	*tcd*B negative (N = 863)	*P*
Age, median (95% CI^a^)	1.6 (1.4–2.2)	3.8 (3.3–4.5)	< 0.01
Age group (N)			
0 (219)			
Inpatient	12	140	0.09
Outpatient	11	56	
Subtotal	23	196	
1 (153)			
Inpatient	18	95	0.16
Outpatient	11	29	
Subtotal	29	124	
2–6 (265)			
Inpatient	11	204	< 0.01
Outpatient	15	61	
Subtotal	26	239	
7–12 (171)			
Inpatient	7	137	0.42
Outpatient	3	34	
Subtotal	10	161	
13–18 (152)			
Inpatient	9	125	0.36
Outpatient	0	27	
Subtotal	9	143	
Total (960)			
Inpatient	57	674	< 0.01
Outpatient	40	189	
Subtotal	97	863	
Sex			0.64
Male	54	502	
Female	43	361	
Length of stay at testing, median (95% CI)	5 (3.6–6.0)	4 (4.0–5.0)	0.807
30-day mortality	2	10	0.346
Other gastrointestinal pathogens			
Detected	30	295	0.52
Not detected	67	568	

^a^Confidence interval (CI)

No *tcd*B was detected in neonates (0/13). They were admitted to the neonatal intensive care unit (NICU) and administered antibiotics. Their mean length of stay was 5.0 days from the day of testing. No other diarrheal pathogens were detected in these neonates. While the youngest *tcd*B-positive infant was a 4-month-old, the *tcd*B positivity rate among infants aged 1 month to 1 year was 11.2% (23/206).

**Fig. 1:**
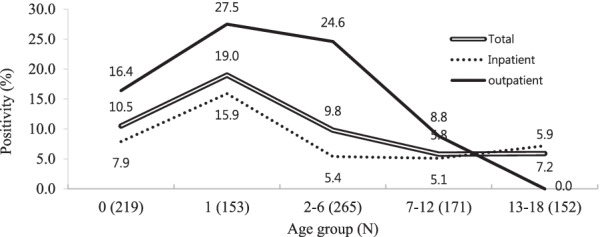
*Clostridioides difficile* toxin B positivity detected by multiplex PCR of indicated age groups

The *tcd*B positivity peaked at 1 year of age (29/153, 19.0%) and was inversely correlated with age. In children aged 2–6 years, the positivity rate dropped to 9.8%, and this incidence decreased in the group aged 7–12 years (5.8%) and 13–18 years (5.9%) (Fig. 1). The *tcdB* positivity rates were higher in outpatients than in inpatients, except in the 13–18 years group (*P* < 0.001).

Among inpatients, the difference in hospital length of stay between *tcd*B positivity and *tcd*B negativity was not statistically significant. Sex and 30-day mortality were not related to *tcd*B positivity. None of the underlying diseases were related to *tcd*B positivity. We categorized the underlying diseases into eight groups, and none of the odds ratios in each group reached statistical significance (data not shown).

### Presence of concomitant gastrointestinal pathogens

Other gastrointestinal pathogens were detected in the stool samples from 325 patients (33.8%) using mPCR. *Clostridium perfringens* 32.6%, norovirus 20.9%, *Campylobacter* spp. 14.5%, and *Salmonella* spp. 10.2% were detected. Among them, 30 (9.2%) had *C. difficile* and other pathogens simultaneously. In 635 patients, no proven etiology of diarrhea was detected. Among them, 67 patients (9.0%) were *tcd*B*-*positive. Altogether, the presence of concomitant gastrointestinal pathogens did not affect the *tcd*B positivity rate (*P* = 0.52).

### Antibiotic exposure

Antibiotic exposure did not increase *tcd*B positivity. The odds ratio of antibiotics in the antibiotic-treated group (N = 541) compared to that in the antibiotic-naïve group (N = 419) was 0.44 (95% confidence interval–CI: 0.28–0.67; *P* < 0.001). Interestingly, when we stratified the groups by age, the *tcd*B positivity was inversely proportional to antibiotic exposure in those under 7 years of age. The odds ratios of the groups over 7 years of age were not statistically significant (Table 2).

**Table 2:** Odds ratio of antibiotics exposed group compared to antibiotics naïve group stratified by age

Age (years)	Odds ratio	95% CI	*P* value
0	0.24	0.08–0.66	<0.01
1	0.35	0.14–0.79	0.01
2–6	0.38	0.17–0.87	0.02
7–12	1.35	0.34–5.41	0.67
13–18	1.23	0.32–4.78	0.76
Total	0.44	0.28–0.68	<0.01

^a^Confidence interval (CI)

### Diagnosis and treatment of *C. difficile* infection

A review of the electronic medical records revealed that a total of 22 patients (22/960, 2.3%) were clinically diagnosed with CDI and treated with metronidazole or oral vancomycin, but nine had no proven existence of *C. difficile tcd*B (data not shown).

## Discussion

In neonates, *C. difficile* frequently colonizes the gastrointestinal tract without causing disease since colonization rates are reportedly 25–36% at 1 month of age [7]. Al-Jumaili et al. [10] found that the isolation rate of toxigenic *C. difficile* increased progressively with infant age, from 7% at birth to 100% by 26–35 days. Unlike previous reports, we did not find any toxigenic *C. difficile* in newborns and infants under 4 months of age. In this study, antibiotics were administered to all 13 patients in the NICU and 74% of patients under 4 months of age. In neonates, antibiotic administration has been reported to delay *C. difficile* colonization for at least 2 months [11]. This may explain why neither neonates nor infants of up to 3 months of age had detectable *C. difficile* in this study. The odds ratio, which was statistically significant in age group 0, including neonates, indicated that antibiotic usage does indeed delay *C. difficile* colonization (Table 2).

Larson et al. [12] surveyed three postnatal wards and reported a positivity rate of 2–52%, and their difference was statistically significant. They also found epidemiological clusters in ward environments. They suspected a nosocomial spreading, which caused the high prevalence in previous studies. Hospitals systematically develop many infection control measures, such as hand hygiene and standard precautions, which may result in a lower acquisition rate in neonates. Rousseau et al. [13] classified the acquisition period into the neonatal phase (early) and infant stage (4–6 months, late). Our youngest toxigenic *C. difficile tcd*B-positive infant was 4 months old; therefore, the subject would have been included in the “late acquisition” group in Rousseau’s study. Late acquisition was reportedly caused by modifications in the gut microbiota composition during a variable food trial.

The high colonization rate of *C. difficile* in infants could result from the commensal microbiota in the pre-weaning period, dominated by *Bifidobacterium* spp. and *Lactobacillus* spp., which are more permissive to colonization [14]. After solid food intake, the microbiota is similar to that of adults, dominated by *Bacteroidetes* and *Firmicutes* spp. According to a longitudinal observation of the gut microbiome analyzed by 16 S rRNA gene sequencing from an infant, the introduction of solid food at around 4 months resulted in a huge change in the microbiome and the diversity of intestinal microbiota was related to *C. difficile* disappearance [14]. During the observation period, *C. difficile* counts varied with fluctuations of more than 10^5^ and eventually disappeared at 12 months. This may explain our first detection of *C. difficile tcd*B in a 4-month-old infant.

We observed that antibiotic usage within 30 days did not increase the positivity rate of *C. difficile* (Table 1). The odds ratios of the age groups 1, 2–6, and total indicated that antibiotic usage is inversely related to the *tcd*B positivity rate. Antibiotic use is a major risk factor for adult CDI, and research by Donta and Myers conducted in 1980 using cell culture neutralization assay (CCNA) showed that *C. difficile* toxin could be found in 85% of infants after 14 days of exposure to antibiotics even when the toxin was not detected during antibiotic therapy [15]. However, in our study, only 5% (3/53) were positive for *C. difficile tcd*B 14 days after antibiotic therapy (data not shown). Similar to our results, in a study reported in 2012 using toxigenic *C. difficile* culture method, antibiotic exposure prior to *C. difficile* detection did not cause a difference between positive patients and the overall population [13]. Although the method used in this study showed higher sensitivity than cytotoxic neutralization analysis (CCNA), the positive rate was lower. In a study using CCNA [15], vaginal delivery and breastfeeding were related to high positive rates, with differences in environmental factors thought to be the cause.

Considering how toxigenic *C. difficile* is acquired in these age groups, our study suggests that multiple factors beyond antibiotic usage might affect the positivity rate. Our study was based on a molecular method using fresh stool specimens to detect the *C. difficile tcd*B gene. Molecular testing, which uses cell culture with frozen stool samples, has a higher sensitivity than other methods. Although non-toxigenic *C. difficile* was not included in this study, detecting toxic *C. difficile* is important in clinical settings. Although molecular testing alone is too sensitive and not specific for diagnosing CDI [8], it is an appropriate test to estimate the presence of low concentrations, not infection status by *C. difficile* overgrowth, and production of abundant toxin.

In addition, *C. difficile tcd*B was detected in 10.1% of patients with diarrhea in the population under study (0–18 years of age). In a previous study, *C. difficile* was detected in culture samples of 7.0% of patients with diarrhea and 14.8% of patients without diarrhea between 2 weeks and 16 years of age. Therefore, the *C. difficile* isolation rate in patients without diarrhea was more than 50% higher than that in patients with diarrhea among outpatients [16]. Another study showed no correlation between diarrhea and *C. difficile* colonization rates in infants [17]. Further, a group aged over 8 years had an infection rate of approximately 5%, similar to that of healthy adults [5]. Among children under 15 years of age, Kim et al. [18] reported that 15.6% of the group with diarrhea and 6.7% of the control group had *C. difficile* toxin through cytotoxicity neutralization assay, indicating a higher positivity rate in diarrhea patients. In the group with diarrhea, the possibility of *C. difficile* infection should be considered for some positive patients.

In our results, the clinical characteristics of the *tcd*B-positive group were different from those of adult CDI. Patients in the *C. difficile tcd*B-positive group were younger than those in the negative group. *tcd*B-positivity was found more in outpatients than in inpatients, and length of stay was shorter in this group than in the *tcd*B negative group. Underlying diseases such as neoplasm, hematologic, respiratory, genitourinary disorder, and inflammatory bowel disease were not statistically related to *tcd*B positivity, contrary to adult CDI.

The pediatric patients included in this study were not entirely healthy since they had diarrhea that required hospital visits, during which stool samples were collected and tested for the presence of diarrhea-causing pathogens. Therefore, *tcd*B positivity in this study included both patients with CDI and carriers with non-CDI diarrhea etiology. A limitation of our study is that not all *tcd*B positivity implies colonization. In addition, this study is a retrospective study; there is a possibility that the records were inaccurate, and some data might have been omitted.

In our study, 22 patients were clinically diagnosed with CDI and treated with metronidazole or vancomycin, yet nine of them had no proven existence of *C. difficile tcd*B (data not shown). Moreover, 30.9% (30/97) of *C. difficile tcd*B-positive patients showed positive gastrointestinal pathogens simultaneously. This is in accordance with another study which reported a simultaneous positive rate of > 50% with *C. difficile* [19]. We cannot define the remaining 70% as CDI because we could not exclude all other etiologies.

We noticed that *C. difficile tcd*B positivity was not affected by concomitant gastrointestinal pathogens. This result suggests that most *C. difficile tcd*B*-*positive cases are more likely to be colonization and not CDI. The clinical factors known as risk factors for CDI, such as underlying disease, antibiotic exposure, and hospital administration, did not increase the CD positivity rate in this study. CDI cases were certainly included, but the rate did not appear to be substantial. Therefore, we may cautiously draw a sketch of pediatric CD colonization with this positivity rate rather than CDI.

## Conclusions

*Clostridioides difficile* is thought to be a hospital-associated infectious pathogen. The acquisition in the community seems to have prevailed due to improvements in individual hygiene levels and hospital infection control, at least in the pediatric population. This study demonstrated lower *C. difficile* positivity in the pediatric population than previously reported. Although toxigenic *C. difficile* infection is neither an important nor a common cause of pediatric diarrhea, children can spread it to adults at risk of developing CDI. Therefore, children can act as hidden reservoirs for pathogenic strains in the community. Monitoring of toxigenic *C. difficile* positivity in the pediatric population should be approached as an infection control measure, as well as individual diagnosis.

In stool samples of patients with diarrhea aged 0–18 years, *C. difficile* toxin B positivity by gastrointestinal pathogen mPCR was 10.1%. The presence of concomitant gastrointestinal pathogens was not associated with toxigenic *C. difficile* positivity; therefore, it is difficult to determine whether *C. difficile* is the cause of diarrhea in the pediatric population. The risk of transmission of CDI along with the difficulty of diagnosis should be noted.

## Data Availability

The datasets used and/or analysed during the current study are available from the corresponding author on reasonable request and consultation with the IRB.

## Abbreviations

CDI: *Clostridioides difficile* infection
mPCR: multiplex PCR
NICU: neonatal intensive care unit

## References

[1] Schutze GE, Willoughby RE (2013). *Clostridium difficile* infection in infants and children. Pediatrics.

[2] Lessa FC, Mu Y, Bamberg WM, Beldavs ZG, Dumyati GK, Dunn JR (2015). Burden of *Clostridium difficile* infection in the United States. N Engl J Med.

[3] Spigaglia P, Mastrantonio P (2002). Molecular analysis of the pathogenicity locus and polymorphism in the putative negative regulator of toxin production (TcdC) among *Clostridium difficile* clinical isolates. J Clin Microbiol.

[4] Chitnis AS, Holzbauer SM, Belflower RM, Winston LG, Bamberg WM, Lyons C (2013). Epidemiology of community-associated *Clostridium difficile* infection, 2009 through 2011. JAMA Intern Med.

[5] Miyajima F, Roberts P, Swale A, Price V, Jones M, Horan M (2011). Characterisation and carriage ratio of *Clostridium difficile* strains isolated from a community-dwelling elderly population in the United Kingdom. PLoS One.

[6] Jangi S, Lamont JT (2010). Asymptomatic colonization by *Clostridium difficile* in infants: implications for disease in later life. J Pediatr Gastroenterol Nutr.

[7] Lees E, Miyajima F, Pirmohamed M, Carrol E (2016). The role of *Clostridium difficile* in the paediatric and neonatal gut—a narrative review. Eur J Clin Microbiol Infect Dis.

[8] McDonald LC, Gerding DN, Johnson S, Bakken JS, Carroll KC, Coffin SE (2018). Clinical Practice Guidelines for *Clostridium difficile* Infection in Adults and Children: 2017 Update by the Infectious Diseases Society of America (IDSA) and Society for Healthcare Epidemiology of America (SHEA). Clin Infect Dis.

[9] Sawyer SM, McNeil R, Francis KL, Matskarofski JZ, Patton GC, Bhutta ZA (2019). The age of paediatrics. Lancet Child Adolesc Health.

[10] Al-Jumaili I, Shibley M, Lishman A, Record C (1984). Incidence and origin of *Clostridium difficile* in neonates. J Clin Microbiol.

[11] Holton A, Hall M, Lowes J (1989). Antibiotic exposure delays intestinal colonization by *Clostridium difficile* in the newborn. J Antimicrob Chemother.

[12] Larson H, Barclay F, Honour P, Hill I (1982). Epidemiology of *Clostridium difficile* in infants. J Infect Dis.

[13] Rousseau C, Poilane I, De Pontual L, Maherault A-C, Le Monnier A, Collignon A (2012). *Clostridium difficile* carriage in healthy infants in the community: a potential reservoir for pathogenic strains. Clin Infect Dis.

[14] Davis MY, Zhang H, Brannan LE, Carman RJ, Boone JH (2016). Rapid change of fecal microbiome and disappearance of *Clostridium difficile* in a colonized infant after transition from breast milk to cow milk. Microbiome.

[15] Donta ST, Myers MG (1982). *Clostridium difficile* toxin in asymptomatic neonates. J Pediatr.

[16] Boenning DA, Fleisher GR, Campos JM, Hulkower CW, Quinlan RW (1982). *Clostridium difficile* in a pediatric outpatient population. Pediatr Infect Dis.

[17] Holst E, Helin I, Mårdh P-A (1981). Recovery of *Clostridium difficile* from children. Scand J Infect Dis.

[18] Kim K, Suh I-S, Kim JM, Kim CW, Cho Y-J (1989). Etiology of childhood diarrhea in Korea. J Clin Microbiol.

[19] Valentini D, Vittucci A, Grandin A, Tozzi A, Russo C, Onori M (2013). Coinfection in acute gastroenteritis predicts a more severe clinical course in children. Eur J Clin Microbiol Infect Dis.

